# On the Functions of the Cerebral Nerves and the Sympathetic

**Published:** 1841-04

**Authors:** 


### BRITISH AND FOREIGN MEDICAL REVIEW,

FOR APRIL, 1841.

### PART FIRST.

### Analytical and Critical Reviews.

### Art. I.

G. Valentin *de Functionibus Nervorum Cerebralium et Nervi Sympathici.* Libri Quatuor.—*Bernæ,* 1839. 4to, pp. 161.

*On the Functions of the Cerebral Nerves and the Sympathetic.* By G. Valentin. In Four Books.—*Berne,* 1839.

Any science is under great obligations to those who, during the period of its most rapid advancement, are not seduced by the novelties that daily crowd upon their notice into the paths of brilliant speculation; but who devote their time and talents to bring together what has been put forth in various and distant quarters as ascertained truth; and who,—not indiscriminately heaping up the materials they have collected, but themselves carefully examining their several merits, deciding by newly-sought evidence between conflicting statements, and filling up by their own enquiries the numerous small gaps that are left between the erections of others,—aim at presenting a systematic and harmonious view of our *positive* knowledge on the subject. And in no science is such a labour more needed than in physiology. We have often heretofore noticed the peculiar uncertainty attending the results of experiment, and the hesitation that must always be felt in drawing inferences from observation, in this science; this want of definiteness arising, there is good reason to believe, not from anything less certain in the *laws* of vitality than in those of other departments of philosophy, but from the peculiar manner in which those laws are brought to bear upon one another, and the many perturbations which arise from their mingled operation. Physiology, then, more than any other department of science, needs such an occasional review; and the qualifications for success, needed by him who shall undertake it, are scarcely less,—in some respects much greater,—than those required for pursuing the path of original research. What these respectively are, we have no doubt that our intelligent readers will easily perceive. They are possessed in a very high degree by the author of the treatise before us. Already well known as an original discoverer in some of the most intricate departments of his science, he has applied his talents most laboriously, and we think successfully, to the determination of our positive knowledge of neurology; and though his volume contains no one brilliant discovery, and not many original speculations, there are few which, in our estimation, can rank above it in real value. We shall, therefore, offer to our readers a tolerably full analysis of its contents, for the information of those who are inclined to be satisfied with such a concise view, and for the purpose of inciting those who make physiology a main object of pursuit, to possess themselves of the original.

This physiological treatise is to be regarded as a continuation of the anatomical description of the nerves, published three years previously. A third part will contain an account of the evolution of the peripheral portion of the nervous system; and the author hopes then to be able to treat of the development of the central organs in the same detail.

In the first book, after a chapter devoted to the *Lex Belliana*, the functions of the cerebral nerves are discussed. The second book contains the physiology of the sympathetic nerve. In the third book are considered the laws which govern the actions of the peripheral nerves. And in the fourth is discussed the influence of the nerves on the several functions, organic and animal. Throughout the whole, a very complete knowledge of the literature of the subject is evinced; and the references to the writings of others are extremely copious and valuable, yet are so arranged as not to interfere with the perusal of the text. What is sufficiently well ascertained to be physiological truth is carefully separated from that which, though probable in itself, requires confirmation; the author's own theories are stated quite distinctly from his experimental results; and the general conclusions may be always readily apprehended. When discussing the special functions of the nerves, the author first points out the information to be derived from anatomical observation of the origin and distribution of each. He then describes the method in which he derived it, states the results of his experiments, and compares them with those obtained by others, of which a general review is given. And lastly, the evidence obtained from pathological phenomena in man is considered.

CHAP. I. *On the Law of Bell.* The general fact that the posterior roots are sensory and the anterior motor is considered by Valentin to have been experimentally established beyond all question; and he regards the observation of pathological phenomena to be here quite subordinate in value to well-conducted experiments, since a natural disease is not a simple but a composite and varied experiment, and its results are liable to be influenced by a great variety of disturbing causes. Where pathological phenomena coincide with the facts ascertained by physiological investigation, they may be accepted as confirmatory of these; but if they should seem to run counter to them, they are not to be set down as altogether disproving them, since a more complete knowledge of their conditions would probably enable us to account for what seems anomalous. We are not yet in a condition to say, for example, what amount of integrity of the nervous tissue is compatible with the performance of its functions; and a mere statement that it is indurated or softened is by no means sufficient to establish its loss of power. With these remarks we pretty much agree; but, applying them to the case in question, we must own that we consider our knowledge of the respective functions of the two roots of the spinal nerves to be much more positive than that of the offices of the divisions of the cord with which they are connected.

That the transmission of impressions is always centripetal in the sensory nerves and centrifugal in the motor appears to be an equally well-established fact. Recent microscopic examination has also demonstrated that which was long ago suspected,—that the individual nervous fibres run with perfect distinctness from their origin to their termination; and that, though an interchange of fibres, producing an anastomosis, often takes place among the trunks, there is no such an anastomosis in the individual fibres. Hence it is probable that every fibre maintains its peculiar endowments through its whole course. This doctrine is the foundation of the entire modern system of nervous physiology.

It is well known that Bell and others have laid great stress on the presence of ganglia on the posterior roots, and other points of anatomical correspondence, in their comparison of the cerebral with the spinal nerves; but no evidence drawn from such anatomical examination can, in Valentin's opinion, be fairly put in competition with that derived from physiological experiments,—although confirmatory of it, should it correspond. For this he states his reasons in detail. Nor can the ultimate distribution of the nerves be alone relied on. For though in general the nerves which proceed to the skin or mucous surfaces are sensory, and those which are sent to the muscles are motor, there are many exceptions; and the absence of general sensibility in the nerves of special sense could not be thus predicated. The primitive fibrils of the nerves appear to be the conductors of impressions; and these are isolated from each other by sheaths, which are thicker in the nervous trunks than in the central organs, as if to provide for their more complete segregation in the discharge of their functions. In the opinion of Valentin it is in the gray matter alone that motor changes originate, and that sensory impressions are received. A few remarks on the nervous system of invertebrated animals are appended to this chapter. They contain some experiments on a lobster, the general result of which is confirmatory of Dr. Carpenter's views as to the respective functions of the cephalic and ventral ganglia. Valentin considers that there is no physiological evidence of the motor and sensory functions of the two strands of the cord, as attributed to them by Newport, and, in a different way, by Grant.

CHAP. II. *Olfactory Nerve.* In this chapter, the account of the individual nerves is commenced with the first pair. This, it is stated, possesses in itself no motor properties; nor is it an excitor of motion through the nervous centres; for no contraction of muscles can be produced by irritating either end of it when divided. It is not a nerve of common sensation; for animals do not exhibit any sign of pain when it is subjected to any kind of irritation. The division of the nerve, or the destruction of the olfactive ganglia, does not seem to inconvenience them materially. They take their food, move with their accustomed agility, and exhibit the usual appetites of their kind. The common sensibility of the parts contained in the olfactive organ is in no degree impaired, as is shown by the effect of irritating vapours. But the animals are destitute of the sense of smell, as is shown by the mode in which these vapours affect them. At first they appear indifferent to their presence; and then suddenly and vehemently avoid them, as soon as the Schneiderian membrane becomes irritated. Moreover, if two dogs with the eyes bandaged, one having the olfactory nerves and ganglia sound, and the other having had them destroyed, are brought into the neighbourhood of the dead body of an animal, the former will examine it by his smell; whilst the latter, even if he touches it, pays no attention to it. This experiment Valentin states that he has repeated several times, and always with the same results. Further, observation shows that sensibility to irritants—such as snuff, and acuteness of the power of smell, bear no constant proportion to one another; and there is ample pathological evidence that the want of this sense is connected with some morbid condition of the olfactory nerves or ganglia.

CHAP. III. *Optic Nerve.* No chemical or mechanical stimulus of this nerve produces *direct* muscular motion; but a reflected motion is excited through the third pair, as is proved by the contraction of the pupil when the nerve is divided, this not taking place if the central organs be removed. The contracted state of the pupil usually soon disappears. That common sensibility is retained when the functions of the optic nerve are completely destroyed is well known; as is also the fact that division of it puts an end to the power of vision. Valentin states that, although the optic nerve may, like other nerves, be in appearance completely regenerated, he has never been able to obtain any evidence that the power of sight has been in the least degree recovered. He remarks that animals suddenly made blind exhibit great mental disturbance, and perform many unaccustomed movements. The complete destruction of the sense of vision, by division of the optic nerve, is easily proved by experiment. The eyelids are not closed when a strong light is suddenly directed on the eye, as is the case when the eye is sensible to it. Again, the state of the pupil is not affected when a strong light is made to impinge upon the retina, except, however, in the following cases: 1. Where the division of the nerve is incomplete, imitating a partial amaurosis in man. 2. Where the rays of light impinge on the iris, in which case they cause contraction of the pupil by reflex action through its own afferent nerves; and Professor Valentin remarks that this irritability of the iris seems much increased by section of the optic nerve. 3. Where the solar rays fall on the eye for a period sufficient to affect its temperature, so that the nerves of common sensation may serve as excitors to the reflex action. (This explanation of the contractility of the pupil in eyes entirely amaurotic has been already suggested by Mr. Grainger.) 4. Where the pupil of the sound eye undergoes alteration in form, so that one stimulus excites the motor nerves of both eyes. This is often seen in amaurotic patients. We see no reason, however, to depart from the view proposed by Dr. M. Hall and Mr. Grainger, that the activity of the pupil in some cases of amaurosis may be owing to the completeness of the nervous circle requisite for reflex action, the disease which obstructs vision being higher up in the encephalon. On this point Professor Valentin's experiments do not bear, as in all of them the optic nerve itself was divided. He justly remarks that the contraction of the pupil is by no means the only reflex action excited through the optic nerve; but that the orbicularis is thrown into action by the incidence of light upon the retina when in an irritable state, as is so peculiarly seen in scrofulous ophthalmia; and that the involuntary movement of the ball (generally in a horizontal direction), which is so often noticed in cases of imperfect sight, is to be attributed to the same cause. Morbid changes are sometimes observed to take place in eyes whose optic nerve has been divided; but these are by no means so constant or extensive as when the fifth pair is paralyzed; and they may not improbably be attributed to the injury occasioned by the operation itself to the parts within the orbit. It is well known that eyes which have been amaurotic for many years preserve their usual appearance; but where the amaurosis is complete, the texture of the retina is changed. It appears as a fibrous membrane, composed of white cylindrical threads; and does not exhibit any appearance of granules, of nucleated globules, or of primitive nervous fibrils; and the yellow spot of Soemmering becomes paler, and is at last undistinguishable. But if a very slight degree of sensibility to light remain, these changes are much less decided. It is well known that when the sight is destroyed by a disease or injury which prevents the passage of light through the pupil, the whole eye becomes more or less atrophied; and the retina and optic nerve are found after death (if the morbid condition have lasted sufficiently long) to have lost their characteristic structure. It seems evident, then, that the continuance of their functional operations is a necessary condition of the maintenance of their normal organization; and we can very well understand that this should be the case, from the analogy of other parts of the system.

Our author next proceeds to discuss, with considerable minuteness, the phenomena of *subjective* vision, as distinguished from *objective;* the latter being excited by the impressions of external objects on the retina, the former by some internal affections of the nervous system. Subjective vision may originate from changes either in the nervous centres, as in delirium, or in the vivid conceptions of the memory; or from changes in the optic nerve or retina, as when stimuli directly affect these parts. Regarding the causes of the different images thus produced, we have very little knowledge; yet it may be noticed that particular effects commonly succeed the application of certain stimuli. Sometimes the interior of the bulb itself may be represented to the mind in this way, as in the celebrated experiment of Purkinje, in which the circulation of the blood in the vascular plexus of the retina may be made the subject of observation. Professor Valentin gives a minute description of the appearances he can himself produce in one of his eyes, which he considers as a representation of certain parts in its interior. As an instance of subjective vision originating in the central organs, he mentions that he has frequently, after devoting several hours to the use of the microscope, and especially when he has been much interested in the object of his investigation, perceived during sleep (but not he thinks while dreaming) most accurate and beautiful representations of the objects which he had been observing. In some cases it would appear likely that visual impressions are excited by external causes, which are made to assume a particular character in the mind by the influence of memory and fancy. And it is, perhaps, owing to the want of the connexion of such impressions with previous percéptions, that persons born blind, and sometimes those who have once enjoyed the power of vision, but have long been destitute of it, do not seem to have any visual conceptions even when dreaming. In this respect, then, the case does not resemble that of a person who has lost a limb by amputation, and is subject to a perception of its pains and movements; and the observation tends to confirm the marked distinction between the perceptions acquired from common and from the special sensations, which has latterly been much insisted on, both by metaphysical and physiological writers.

CHAP. IV. *Oculo-motor Nerve.* The experiments hitherto performed on this nerve are not satisfactory in regard to the degree of sensibility it possesses. From those of Valentin it appears that pain is felt when the nerve is divided; but that this is not so great as that occasioned by section of the fifth pair. At the moment of section, the pupil of the affected eye, and frequently also that of the other, is strongly contracted. If the animal lives long enough after the operation (which, on account of the almost inevitable division of the carotid artery, is seldom the case,) the pupil becomes again dilated and remains paralyzed. No amount of light thrown on the retina or the iris occasions its contraction; nor does the iris ever move by sympathy with that of the other eye. Hence it follows that the oculo-motor is the chief source of the movements of the iris; but it will be hereafter shown not to be the only spring. The other functions of the nerve may be best examined by removing the brain, and applying mechanical or chemical stimuli to the trunk. Its influence on the pupil then becomes very evident. The eyeball is at the same time rapidly rotated inwards, more rarely inwards and upwards, and more rarely still downwards. Motion of the upper lid is very seldom seen; but this is probably due to the injury which the fibres of the levator palpebræ have sustained by the operation. The general motor functions of this nerve are sufficiently well established by cases of paralysis in man. When the distribution of the branches of this nerve is considered, in connexion with the actions of the different muscles it supplies, it will appear probable that they possess various endowments. The superior branch derives additional sensory fibres from the naso-ciliary branch of the ophthalmic (fifth pair); and in this manner the sensory endowments of this division of it are probably increased. The sensory endowments of the seventh pair will be shown to be entirely derived from anastomosis with the fifth and cervical nerves; and it will be recollected that a similar connexion appears to exist between the anterior and posterior roots of the spinal nerves. (See Br. and For. Med. Rev., vol. IX., p. 547.) This superior branch of the oculo-motor supplies the superior rectus and levator palpebræ; the action of which appears to be of a purely voluntary character. The inferior branch supplies the internal and inferior recti and the inferior oblique, and also the iris. Now the motions of the iris are altogether automatic or reflex. Some have regarded them as in a degree voluntary; but the so-called voluntary contraction of the pupil is always connected with some action of the muscles of the eyeball. The inferior oblique appears to have an almost solely involuntary action; by it is performed the rotation of the ball upwards and inwards, which is observed to take place during sleep, the internal rectus perhaps assisting. The pupil is at the same time contracted; and this also happens if each eyeball be rotated, by an effort, towards the inner canthus. But the pupil is not contracted when the eye is voluntarily rotated upwards; so that the action of the iris is evidently dependent upon the inferior rather than upon the superior branch of the oculo-motor; and it is only by such an impulse transmitted along the former, as will also excite the action of the muscles of the ball which it supplies, that the so-called voluntary movement of the pupil can be produced. It is apparently by this branch only that the reflex actions are performed, which are excited by impressions transmitted through the optic nerve and fifth pair. This view of the somewhat antagonistic functions of the two branches is supported by the symmetrical distribution of the muscles. The rectus externus, supplied by the sixth pair; the rectus superior, supplied by the superior branch of the third; and the obliquus superior, supplied by the fourth pair,—are all, in Professor Valentin's estimation, purely voluntary muscles. Whilst the rectus internus, rectus inferior, and obliquus inferior, supplied by the inferior branch of the third, are more or less automatic in their action, the iris being entirely so. These may be likened to the flexors, whilst the three voluntary muscles perform the part of extensors.

CHAP. V. *Pathetic Nerve.* The course of this nerve within the cranium renders experiment upon it difficult; and it cannot be satisfactorily ascertained whether any pain is felt by the animal when it is divided. Of its motor action there can be no doubt. This may be most readily demonstrated by removing the brain and irritating the trunk of the nerve. The eyeball is then rotated downwards. The character of this movement is discussed along with that of the sixth pair, to which we shall next proceed, with the view of bringing together all which relates to the motions of the eyeball.

CHAP. VII. *Abductor Nerve.* Experiments upon animals and observation of cases of paralysis in man leave no doubt that the sixth pair of nerves is chiefly if not entirely motor, and that its action is confined to the external rectus. The course of this nerve within the cranium renders it almost impossible to determine by experiment whether or not it contains any sensory fibres; but if any exist in it, they are probably very few in number. The muscle which this nerve supplies appears to be entirely under the control of the will.

The following classification of the movements of the eyeballs and the succeeding remarks are, we think, of sufficient importance to merit being given in full:

"The regular conjoint movements of the eyeballs are rather *harmonic* than *symmetrical.* The varieties which they present may be reduced to the following classes: 1. *One eye is rotated inwards, the other outwards.* Here the internal rectus of one eye and the external rectus of the other are put in action. 2. *Both eyeballs are elevated* by the contraction of the superior recti. 3. *Both are depressed.* This is at first effected by the conjoint action of the inferior recti alone, but if the movement go beyond a certain point the eyes are rotated inwards, showing that the internal recti are also in action. 4. *Both are drawn directly inwards and downwards* by the action of the internal rectus, joined either with the inferior rectus or the superior oblique. 5. *One eye is rolled upwards and outwards, the other upwards and inwards.* In this case the rectus superior of both eyes acts with the external rectus of one and the internal rectus of the other. 6. *One eye is drawn downwards and outwards, the other downwards and inwards;* the inferior rectus acting in both with the external rectus of one and the internal of the other. All these movements may be voluntarily performed by man; there are two others which he cannot directly perform by an act of the will, the one occurring during sleep, the other when an object is brought very near the eyes. In the first of these cases, 7. *Both eyeballs are rotated upwards and inwards* by the action of the inferior oblique, assisted perhaps by the internal rectus. In the other, 8. *Each eye is directed inwards* by the interior rectus, conjointly with the superior or inferior rectus; in the latter case it is most complete.

"Hence it follows: 1. There is one movement amongst all these, which is at the same time harmonic and symmetrical, the elevation of the eyeball by the superior rectus. The depression of it by the inferior rectus is so in the first instance; but when the eyes are rotated inwards the motion loses its harmony, though it is still symmetrical. 2. All the regular motions of the ball which are harmonic, but asymmetrical (as in class 1), are effected by a contraction of one of the muscles formerly specified as voluntary, and one which is partly automatic. 3. Two voluntary muscles whose actions will produce want of harmony between the eyes (such as the superior rectus and superior oblique), cannot be made to contract together. 4. Two automatic muscles whose action destroys the harmony of the eyes may be contracted together, as in classes 7 and 8; in the first case the action cannot be voluntarily produced; in the second it may be, but becomes more decided when the internal rectus is assisted by the inferior than when the action of the superior is conjoined with it. [We are disposed to question whether this movement is voluntary; it seems to us to require an object to which the eyes may be directed.] 5. Voluntary squinting (lusciatio) is performed by those muscles only whose action is chiefly automatic; as a result of disease (when not depending upon lesion of the iris or retina) it is mostly caused by the automatic muscles.

"From these data, the following account of the muscles of the eyeball may be given. In each eye of man and animals there are two opposed problems to be reconciled. On the one hand, *symmetrical* muscles should exist, as in other parts of the body. On the other, the movements should not be symmetrical but *harmonic*. Again, some of the movements of the eyeball must be voluntary, whilst others depend upon various affections of the retina. To the former objects the external muscular circle (consisting of the rectus superior and externus and the obliquus superior) is destined; and to the latter the internal muscular circle (comprehending the rectus inferior and internus and the obliquus inferior). The pupil is dilated by the voluntary circle [we do not see the evidence of this]; and contracted by the automatic. The former corresponds with the extensors, and the latter with the flexors, and is hence more readily caused to contract, both in health and disease. But the several muscles belonging to one circle are respectively opposed by others in the second circle; and harmonic motion always results from the action of a muscle of the voluntary circle on one side and of the automatic on the other. Hence we see why this peculiar disposition of the muscles should constantly exist. Nor is the arrangement of the nerves less elucidated by this view. It is evident that the automatic muscles should derive their power from the same source as that which produces contraction of the pupil, and that the external rectus and superior oblique should have their own nerves. The supply of the superior rectus from the third pair is also accounted for, its action being harmonious with that of the levator palpebræ, and these being the only two muscles of the voluntary circle whose action is accompanied by a dilated state of the pupil, and which, therefore, would require to be connected with the same system of nerves as that which governs the iris." (pp. 30-1.)

Chap. vi. *Fifth Pair.* When the trunk of this nerve is divided in the living animal (as Prof. Valentin has seen very successfully accomplished by Magendie) evident signs of acute pain are given. After the incision has been made through the skin, the animal remains quiet until this nerve is touched, and when it is pressed or divided it utters doleful cries, which continue for some time, showing the painful effect of the irritated state of the cut extremity. The common sensibility of all the parts supplied by this nerve is entirely destroyed on the affected side. The jaw does not hang loosely, because it is partly kept up by the muscles of the other side; but it falls in a slight degree, and its movements are seen, when carefully observed, to be somewhat oblique. If the trunk be divided on each side, the whole head is deprived of sensibility, and the animal caries it in a curious vacillating manner, as if it where a foreign body. If the anterior branch only be divided, all the parts supplied by it are found to have lost their sensibility, but their motions are unimpaired. The same is the case with regard to the second branch. But if the third branch be divided, the jaw droops, as just stated; and irritation of the trunk will produce convulsive contraction of the masticator muscles. When the whole nerve or its anterior branch is divided in rabbits, the pupil is extremely contracted, and remains immoveable. But in dogs and cats and pigeons it is dilated. The pupil of the other eye is scarcely affected, or if its dimensions be changed, it soon returns to its natural state. The changes in the eyeball consequent upon section of the fifth pair, which have been described by Magendie, have been also observed by Valentin. He states that their commencement may be noticed before the lapse of twenty-four hours after the operation. All these conclusions are confirmed by pathological observations on the human subject.

In regard to the *ophthalmic* branch, all experiments and pathological observations concur in attributing to it sensory endowments only. The only apparent exception is in the case of the naso-ciliary branch, since there is good reason to believe that the long root of the ciliary ganglion and the long ciliary nerves possess motor powers. But these, as will be hereafter seen, are derived from another source,—the sympathetic nerve. The same may be said of the *superior maxillary* branch considered in itself; but its connexions with other nerves, through the spheno-palatine ganglion and its anastomosing twigs, introduce other kinds of primitive fibres into it. These connexions are minutely described by Valentin.

The *inferior maxillary* branch is the only one which possesses motor as well as sensory endowments from its origin; but its different subdivisions possess these endowments in varying proportions, as Valentin shows in much detail. Sir C. Bell's views of the special character of the motor portion as a nerve of mastication, are fully borne out by his researches. The function of the lingual branch will be discussed with that of the glosso-pharyngeal.

The general analogy of the fifth pair with the spinal nerves is sufficiently evident. Yet there are several important points of difference. For in the spinal nerves, after the conjunction of the two roots, the branches into which the trunk divides have mixed endowments, both the anterior and posterior spinal nerves being at the same time motor and sensory. But in the fifth pair, the anterior divisions (corresponding with the anterior spinal branches) are purely sensory, and remain so until they acquire motor properties by inosculation with the facial. Moreover the branches of the third division, which in the first instance possess a mixed function, often lose their motor fibres in their course, and become entirely sensory. The three branches of the sensory division are united by a plexus, as are also the second and third by themselves. This plexiform arrangement finds no analogy in the spinal nerves; the junction of whose anterior and posterior roots is only represented in the fifth pair by the imperfect union of the sensory and motor divisions of the third branch in the plexus of Santorini and Girard. All the subdivisions of this branch are from their commencement, of a mixed function; and it is here therefore, and not with the whole of the fifth pair, that we are to regard the analogy with the spinal nerves as existing.

CHAP. VIII. *Facial Nerve.* That this nerve is solely one of motion, as maintained by Bell, has been more lately denied by Arnold and others, who have maintained that it both possesses motor endowments, and arises by a double root. The latter assertion is quite fallacious; the former can only be determined by experiment. Valentin found that the only way to divide the seventh pair within the cranium, without so much disturbing the roots of the other nerves as to render the experiment useless, was to remove the whole posterior part of the bony covering. In this way he ascertained that the animal gave no sign of pain when the facial nerve was touched or divided; although it uttered violent cries when the roots of the vagus were touched. By this it was demonstrated that the facial at its origin contains no sensory fibres. The numerous anastomoses which it undergoes before its exit from the stylo-mastoid foramen are minutely described by Valentin, who traces microscopically the course of the individual fibres from one nerve into another; but his details would be out of place here, though no cultivator of physiological anatomy ought to neglect them. That the facial nerve, when about to be distributed to the muscles of the face, possesses some degree of sensibility, seems to have been allowed by Bell, and has been pointed out by many subsequent experimenters; and some have attributed it to anastomosis with other sensory nerves, especially the fifth. Eschricht and Lund endeavoured to determine this by laying bare the portio dura at its exit from the stylo-mastoid foramen, and then dividing the trigeminus at the base of the skull. It was then found that the sensibility of the parts anterior to the auditory foramen was entirely extinguished, whilst posteriorly it remained. It may be inferred, therefore, that the sensibility observed in the portio dura depends on anastomosis with the fifth; that which remained in the back part of the head being derived from the cervical nerves. Panizza also proved that, if the seventh pair be cut in a horse in front of the parotid gland, signs of pain are given; but that if the gland be dissected from over it, so that it may be divided before its anastomosis with the fifth, no indication of sensibility is then given. Several such anastomoses appear to take place; the degree of each varying in different animals, so that it is difficult to lay down any general propositions as to the sensibility of the several branches of the vagus. However, the fact that whatever sensibility it may possess is derived by its anastomoses with other nerves appears fully established. The account given by Valentin of its motor functions corresponds almost entirely with that of Bell. He considers it now beyond a doubt that it is the general motor nerve of the face, that the expression of the countenance depends upon its integrity, and that all the respiratory movements of the mouth and nose are performed through this channel; those of mastication, on the other hand, being entirely due to the third division of the fifth pair. His testimony is valuable, being evidently the result of a very detailed and careful examination of the question; but there is no such novelty in the evidence adduced as to render it desirable for us to present it more fully in this place.

CHAP. IX. *Auditory Nerve.* The functions of this nerve are easily determined by anatomical examination of its distribution, and observation of pathological phenomena. Atrophy or lesion of the trunk destroys the sense of hearing; whilst irritation of it produces *subjective* phenomena, analogous to those of vision. From experiments made upon this nerve before it leaves the cranial cavity, it appears satisfactorily ascertained that it has no motor power whatever, and that it is not endowed with common sensibility, the animals not giving any sign of pain when it is irritated or divided. Microscopic examination of this nerve clearly indicates its intermediate character between the anterior and posterior sensory nerves issuing from the cranium, namely, the olfactive and optic, and the glosso-pharyngeal. Nucleated globules are not found in its origin, but they may be traced in its passage through the meatus auditorius, and in the labyrinth. The primitive fibres are not so soft as those of the olfactive nerve, nor so slender as those of the optic; but they are softer than those of the glosso-pharyngeal. It forms a plexus with the facial, to which there is no analogy in the optic and olfactive nerves, but to which a similar one exists in the glosso-pharyngeal.

CHAP. X. *Glosso-pharyngeal Nerve.* The functions of this nerve, as ascertained by anatomical research, by experiment, and pathological observation, are very minutely discussed; and, although we are on the whole more inclined to accord with the results obtained by Dr. Reid, and with his inferences from them, than with those of Valentin (which only differ however in subordinate particulars), we think it worth while to follow our author through the heads of his argument. He begins by remarking that, contrary to what has been up to this point observed in the cranial nerves (the third branch of the fifth, which receives the recurrent branch of the superior maxillary being the only exception), the glosso-pharyngeal anastomoses freely with the vagus, hypoglossal, and accessory nerves, before leaving the cranial cavity. The cause of this Prof. Valentin considers to be, that these nerves are not cerebral, but belong to the medulla oblongata, and are thus to be regarded as formed by an *incomplete* metamorphosis of the cervical nerves. A cursory view of the distribution of this nerve would lead to the conclusion, that it possesses motor properties, since several of its branches may be traced into muscles. But a more careful examination shows that these only penetrate the muscles, and really pass on to be distributed to the mucous membrane, forming plexuses with the third branch of the fifth, the facial, and the vagus. Dr. Reid's experiments, which clearly prove that the muscular movements, apparently produced by stimuli acting through this nerve, are, in reality, of a reflex character, and are immediately dependent on the pharyngeal branches of the par vagum, are next adverted to. But the most certain information is that to be derived from examination of the roots of the nerve. According to Müller, the two roots have corresponding endowments with those of the spinal nerves, the one possessing a ganglion (the superior jugular), and the other having the course of its fibres undisturbed. But, argues Valentin, if this be the case, the motor branch, which does not pass through the ganglion, would be the largest of the two; and it is quite clear that if the glosso-pharyngeal possesses any motor properties, they form a small part of its general functions. The petrosal ganglion is not properly accounted for on this theory, Müller attributing it to the anastomosis of a branch of the sympathetic with the glosso-pharyngeal. But, observes Valentin, other anastomoses of equal importance take place elsewhere, without the formation of a ganglion. Stimulation of the roots of the glosso-pharyngeal nerve does not produce those motions of the pharynx which are excited by irritation of the pharyngeal branches themselves, nor does it produce any muscular movement; whence it appears, that no motor fibres originally exist in the nerve. The small amount of the signs of pain exhibited by animals when the trunk of this nerve is irritated or divided, after its exit from the cranium, compared with those which stimulation of the lingual branch of the trigeminus produces, appears to prove that this nerve is by no means entirely one of common sensation, though it certainly contains sensory fibres. These may be introduced into it, however, by the tympanic branch of the trigeminus, and by the branch of the vagus communicating with the petrosal ganglion. But it is probable that some are derived from its own origin. These communicating branches seem to transmit fibres of each nerve to the other; but, on account of their slenderness and their situation, minute examination of them is very difficult.

The important question next arises, whether the glosso-pharyngeal is the nerve of taste. The experiments to be made for this purpose must be performed on the nerve after its exit from the cranium; for even if the animal remains sufficiently long alive after the roots of the nerve have been exposed, the disturbance produced by this severe operation is too great to prevent the results of the experiments from being worthy of reliance. For dividing the nerve as near its exit from the cranium as possible, Valentin employs the method of Panizza, whose results he confirms in every particular. He states that, when the division of the nerve has been complete, the sense of taste seems altogether destroyed. Dogs will take decoction of colocynth, and other nauseous ingredients mixed in their food, without any appearance of dislike, although after a fast of two days an uninjured dog will reject with disgust that which has but a slight admixture of the same. But if a very small portion only of the nerve on either side remains undivided, enough sense of taste still remains to cause the rejection of the food, and the excitement of reflex motions in the pharynx and upper part of the œsophagus. From such experiments it is concluded by Valentin that the glosso-pharyngeal is the special nerve of taste as well as that which communicates nauseating impressions, and which stimulates the reflex actions resulting from them. In the two latter particulars, the results of Dr. Reid's experiments (which he only quotes at second hand from l'Institut) fully bear out his; but we cannot see that he brings sufficient evidence of the former. Of one very important function of the glosso-pharyngeal nerve,—the conveyance of those impressions from the fauces which excite the reflex movements of deglutition, and which do not appear necessarily to involve sensation, he takes no notice. This has, we think, been fully established by Dr. Reid's experiments; and the small amount of sensibility evinced when the nerve is irritated, is thus fully accounted for, without having recourse to the supposition that the bulk of the nerve is a conductor of special sensory impressions.

The question how far this is also a nerve of taste partly turns upon the function attributed to the lingual branch of the fifth pair, which Valentin next discusses at much length. This he regards as nothing more than a nerve of common sensation; and he states that, after complete section of it on both sides, the sense of taste remains unaffected; whilst, on the other hand, irritation of the trunk evidently causes very severe pain. We do not, however, regard his experiments as by any means sufficient thus to determine the question. They seem to have been performed in ignorance of those of Dr. Alcock, who arrived at very different results, this gentleman inferring from them that the lingual branch of the fifth pair ministers to the sense of taste in the anterior part of the tongue, in which conclusion he is borne out by Dr. J. Reid. If these experiments had been quoted by Valentin, and any fallacies which they may contain had been exposed and corrected, we should have had some ground for coming to a decision upon their respective merits; but at present, the evidence derived from experiment being, to our minds, nearly balanced, the question is rather to be decided by pathological observation, and examination of the anatomical distribution of the glosso-pharyngeal nerve. There is this fallacy, as it appears to us, in Valentin's inference regarding the absence of the gustative sense in the lingual branch of the fifth, that, although it is sufficiently evident that the sense of taste remains in some degree after its division, there is no proof that it is not destroyed in the *anterior portion* of the tongue, which other evidence shows to depend on it for this endowment. Considering, therefore, that the results of physiological experiment are here by no means certain, we cannot agree with our author that pathological phenomena, where they appear to differ from them, are to be thrown to one side. Though the cases in which the gustative sensibility of the anterior part of the tongue has been destroyed, with its tactual sensibility, when there was no reason to suppose that any other than the fifth pair of nerves was involved, are by no means rare, he refers to a few only, on which he thinks that he can throw some doubt; and he seems to think that what is certainly the more frequent case, the loss of common sensibility whilst that of taste still remains, is a powerful argument that the two do not depend on the same nerve. But this proves nothing more to our minds, than that the fibres which serve these two purposes are separate, and have distinct central terminations; for cases are not uncommon in which one of two endowments of a nerve, even when these have a general similarity, as tactual sensibility and sensibility to temperature, or the voluntary and sympathetic motor powers of the seventh pair, is lost without the other being affected. In regard to the anatomical distribution of the glosso-pharyngeal nerve, Valentin asserts that it is not so completely restricted to the root of the tongue as is ordinarily stated; but that it sends a branch forward on each side, somewhat beneath the lateral margin, which inosculates with the fifth, and supplies the edges and inferior surface of the tip. It is quite possible that these may contribute to the gustative sensibility of these parts; but we can by no means agree with Valentin, that the sense of taste is confined to the portion of the tongue thus supplied by the glosso-pharyngeal; and we remain, therefore, in our previous conclusion, that both nerves contribute to it, the lingual branch of the fifth having also a high degree of tactual sensibility, and serving as an excitor to the movements of mastication (when these are involuntary, as in the infant, or so habitual as scarcely to require the constant operation of the will), whilst the glosso-pharyngeal has little common sensibility, but has for its chief function to excite, through the nervous centres, the movements of the pharynx concerned in deglutition, vomiting, &c.

There is one argument on this subject that may be drawn from comparative anatomy, which we do not recollect to have seen noticed,—that of all the senses commonly denominated *special,* that of taste appears most nearly connected with *common* or *general* sensibility; and that some amount of it appears to be possessed by animals which are endowed with no other special sense. Where this is the case, it is evidently connected with the nerve which, issuing from the cephalic ganglion for the supply of the head and anterior part of the body, corresponds with the fifth pair of vertebrate animals. That we may bring together all that relates to the tongue, we shall next proceed to

Chap. xiii. *Hypoglossal Nerve.* That this nerve is principally motor has long been known; but there has been less certainty as to its possession of sensory properties. Indications of pain are given when its trunk is irritated after its exit from the cranium; but these may be supposed to proceed from its anastomosis with the cervical nerves, which probably imparts sensory fibres to it. But in rabbits, each trunk passes out in two bundles through separate orifices, and when the anterior of these is stimulated (in a dead body still irritable) no muscular movements follow, whilst excitement of the posterior bundle causes movement of the tongue. Hence Valentin concludes (without, as it seems to us, that full proof which he might have obtained) that the anterior fasciculus is sensory, and that the hypoglossal is analogous to the spinal nerves; which is further indicated by a slight ganglionic enlargement on the posterior side of its root, which is more perceptible in many of the mammalia than it is in man. The lingual branches of this nerve, however, do not seem to possess any large share of sensory endowment. Valentin seems to consider that *all* the movements of the tongue are dependent upon this nerve; but this we consider by no means established. It often happens in paralysis that the masticatory movements of the tongue are but little affected, when the power of articulation is much injured; and although there can be no question that the lingual branch of the fifth is principally distributed to the surface of the tongue, we do not think that its participation in its movements (which might be *a priori* expected from its origin in the third division of the trigeminus) has been altogether disproved. There is a curious circumstance attending paralysis of this nerve in cases of hemiplegia,—that the hypoglossal is not always palsied on the same side with the facial, but sometimes on the other. This is perhaps due to the origination of the roots of this nerve from near the point at which the pyramids of the medulla oblongata decussate; so that some of its fibres come off without crossing, like those of the spinal nerves; whilst others are transmitted to the contrary side, like those of the higher cerebral.

Chap. xi. *Par Vagum.* We shall give a less full analysis than we otherwise should have done of Valentin's account of the functions of this nerve, since he does not seem to have become acquainted with Dr. Reid's very full, and to our minds very complete and satisfactory, elucidation of them.* His most important original observations, however, with his general conclusions, we shall detail. The result of his first experiment is very unexpected. He states that *at its roots* the par vagum possesses no direct motor power. If these be carefully separated from those of the glosso-pharyngeal, and (which is a matter of some difficulty) from those of the spinal accessory nerve, and be then irritated, no movements of the pharynx, œsophagus, stomach, larynx, trachea, or heart, can be seen in the irritable body of a horse, dog, or rabbit. This experiment requires great care; and various precautions are mentioned, to which those who repeat it should attend. In the living rabbit, a slight irritation of the roots of this nerve appears to produce great pain, although this does not seem to last so long as when the trigeminus is divided. Such irritation produces muscular motions, when the part irritated is in connexion with the nervous centres; but not otherwise. Whence it is clear that these motions are of a reflex character, and that the nerve possesses *in itself* no motor endowments. The spinal accessory nerve, as will hereafter appear, is principally or entirely of a motor character; so that the idea of Arnold and Scarpa, that the par vagum and spinal accessory are together analogous to a spinal nerve; the one answering to the posterior, and the other to the anterior roots, appears sufficiently probable. When the par vagum swells into the jugular ganglion, an interchange of fibres takes place between it and the spinal accessory; but many more fibres pass from the latter to the former, than from the former to the latter. Hence it results that, of the branches into which the par vagum subsequently divides, many enjoy a high degree of motor power; whilst those of the spinal accessory do not appear to possess any great share of sensibility.

In regard to the *pharyngeal* branch, Valentin's opinion coincides with Dr. Reid's, that it is chiefly motor, and that it is in fact *the* motor nerve of the pharynx. He states that it seems also in some degree a nerve of sensation; for, when the glosso-pharyngeals on both sides had been divided, tickling of the fauces produced vomiting. Nevertheless, he observed, as Dr. Reid had done, that the indications of sensibility afforded on pinching the trunk of this branch, were very feeble. The explanation seems to us to consist in this, that the power of conveying such impression as shall excite reflex actions, does not necessarily involve sensibility, as is shown by the action of the lower part of the œsophagus. Valentin states that the anatomical derivation of the pharyngeal branch harmonizes with his view of its chiefly-motor function; Bendz having ascertained that the larger portion of it proceeds from the spinal accessory. As to the endowments of the *laryngeal* branches, also, Valentin's general conclusions agree with Dr. Reid's. He states that the superior laryngeal is especially a sensory nerve, though it may have slight motor power; but that the inferior laryngeal is, on the contrary, almost solely a nerve of motion, although perhaps possessing a slight degree of sensibility. And he seems to think it probable that the movements of the

* See Vol. VIII. p. 264; and Edinb. Med. and Surg. Journal, vols. xlix. and li.

larynx, excited by irritation of the former, are in reality of a reflex character; but this he has not determined, as Dr. Reid has done, by direct experiment. The branches of the vagus which are distributed to the thoracic and abdominal viscera appear to possess both motor and sensory functions. The latter may be inferred from the indications of pain afforded by the animals experimented on, when these branches are irritated.

Before passing to the consideration of the special influence of this nerve on the organs it supplies, Valentin refers to the contraction of the pupil observed after division of the vagus in the neck, in those animals in which the sympathetic anastomoses freely with it, as in the dog, which was long ago observed by Petit, and recently confirmed by Dr. Reid. It seems doubtful whether this effect is produced through the sympathetic or through the vagus. Valentin states that, if the inferior ganglion of the latter be removed, in black rabbits, a decided contraction is produced; though a less manifest effect is produced in white rabbits, by division of both the sympathetic and vagus lower down in the neck. On the other hand, Dr. Reid states it as the result of his experiments on the cat (in which animal the sympathetic and vagus are easily separated in the neck), that section or even compression of the sympathetic instantly produced contraction of the pupil; whilst injury of the par vagum was followed by no such effect. Valentin states that the signs of sensibility given by the cervical portion of the vagus are very indistinct, when the animal has previously suffered much pain; and in this way the contradictory results obtained by experimenters may probably be accounted for.

That the movements of the heart are influenced by the cardiac branches of the vagus, Valentin states that he has obtained distinct evidence; convulsive actions being excited, after the regular movements have ceased, by irritating the cervical portion of the nerve (the result of this experiment, however, being much influenced by the mode of death). But this subject will be further considered when the functions of the sympathetic are enquired into. Passing over the special influence of this nerve on the larynx, as having been much more satisfactorily elucidated by Dr. Reid, we come to the connexion it has with the functions of the respiratory organs. The mucous surface of the trachea and bronchi appears (according to Valentin) to be endowed with some degree of sensibility by branches of the inferior laryngeal; and he remarks that reflex movements may be more easily excited by irritating the surface than by stimulating the trunk of the nerve, which corresponds with what we consider to be a general law of the excito-motor system. He has seen also distinct contractions of the rings of the trachea produced by irritating this trunk in a rabbit; and he thinks it probable that the pulmonary plexus accompanying the subdivisions of the bronchi has a similar influence upon their diameter. No such movements can be seen however in the horse. As to the influence of this nerve on the respiratory process, we find nothing new. All the changes consequent upon section of it he attributes (as we think, with perfect justice) to the insufficient introduction of air into the lungs. But the source of this deficiency he seeks for in the diminution of the opening of the glottis; and he quotes from Arnold the statement that the number of respirations is increased by the operation. Now Dr. Reid has shown that, when free entrance is given to the trachea, the usual effects are still observed; and he attributes them to *diminution* in the number of the respiratory movements, which he considers to be the immediate effect of the partial interruption of communication between the lungs and medulla oblongata, so that the transmission of the necessary stimulus is cut off; and he points out the cause of the idea that these movements are increased in frequency, which many observers have entertained, as he himself did in the first instance.

In order to understand the influence of the par vagum on the function of digestion, Valentin remarks that we must examine three different conditions: 1, its connexion with the movements of the pharynx, œsophagus, and stomach; 2, its influence on the secretion of the gastric juice; 3, the variation which the supply of the stomach with imperfectly arterialised blood will produce. In his opinion, the movements of the alimentary tube are directly affected, the secretion of gastric juice unaltered, and an alteration in the character of the blood indirectly produced, by section of the par vagum. In regard to the first point, his experiments are not sufficiently full, no attempts having been made by him to discover how far the motions of the œsophagus result from a reflected stimulus operating through this nerve. This, indeed, he seems to have taken for granted, in consequence of the distribution of its branches upon the muscular parietes; but from the experiments of Dr. Reid, it would appear that this influence does not go beyond a certain point, which varies in different animals, and that the contractions occurring below this result from the *direct* stimulation of the muscular fibres. Valentin states that the influence of the par vagum on the muscular coat of the stomach, which has been asserted by some experimenters and denied by others, may be most distinctly demonstrated in the rabbit. The experiment is less likely to succeed, if the irritability of the stomach, which is soon lost after death, should be already on the wane, and if the stimulation of the nerve should be performed too high up. The result is seldom wanting, when, in a newly-killed animal, the nerve is irritated in the lower part of the neck, or in the thorax. He further states that the par vagum appears to be the sensory nerve of the stomach, communicating the impressions which produce the sense of hunger or of satiety. Dr. Reid's experiments, however, show that, though this is not improbably true, there is some other source of hunger; as animals who have sustained section of the nerve on both sides will eagerly take food, if they have not received too great a shock from the operation. That when this is the case, however, no feeling of satiety is experienced when the stomach is loaded, Valentin concludes from experiments similar to those of Baglivi, Legallois, and others; but he states that it may be best seen in puppies, who, after the operation, will take three times, and even more, the same quantity of milk as uninjured individuals of the same age, so that the abdomen is greatly distended. He considers it also sufficiently proved that vomiting may take place as a reflex action, excited through the sensory portion of this nerve; although it may be produced also in other ways.

That the par vagum has no direct influence on the secretion of gastric juice, either as to quality or quantity, is the conclusion at which Valentin arrives; not so much, however, from his own experiments, as from those of Arnold, Broughton, and others. On this point, however, the experiments of Dr. Reid have left little to wish for. Its immediate influence upon the function of digestion, therefore, he regards as confined to the movements of the stomach, by which that incorporation of the gastric juice with the whole mass of food is effected, which is essential to its solution. He does not seem to be aware that animals may live for a considerable length of time, and digest enough food for their nourishment, after complete section of the nerves on both sides, as Dr. Reid ascertained. It is probable, however, that the motor influence of the par vagum on the stomach, as on the œsophagus, varies in different animals; and that in some, the movements of the stomach may be nearly independent of it, though capable of being influenced by it. We cannot but regard the independence of the secretion of the gastric juice of any nervous influence as a point now fully established, as far as the par vagum is concerned; and we trust that we shall not be doomed to see Dr. Wilson Philip's often-quoted experiment transcribed from one work to another as an established fact, just as if it had not been proved to be of no value whatever. Verily, there *are* as many false facts as false theories in physiology! On the remainder of the alimentary canal, Valentin does not think that the par vagum has any direct action. No renewal of movement can be observed, upon irritation of the nerve, when it has previously ceased; and absorption, as well as secretion, appear to be properly performed. The inferences deduced from observation of pathological phenomena in man, confirm, so far as they extend, those derived from physiological experiment.

Chap. xii. *Spinal Accessory Nerve.* From the roots of this nerve, as already explained, all the motor properties of the par vagum appear to be derived; no indications of sensation can be obtained by irritating them. When the external branch of this nerve is irritated, the trapezius and sterno-mastoideus are thrown into action, as remarked by several observers, and some, though not violent, indications of pain are given; these, probably, depend on admixture of the sensory fibres derived from the par vagum. In this respect Valentin's observations coincide with those of Dr. Reid, and also in the fact, that section of the nerve does not prevent the trapezius and sterno-mastoideus from sharing in the automatic movements of respiration, which Sir C. Bell imagines to depend upon it.

Chap. xiv. *Phrenic Nerve.* It appears to be sufficiently established that this is the sole motor nerve of the diaphragm, and acts upon it alone. The movements of the heart and stomach, which have been described as resulting from stimulation of this nerve by galvanism, have not been witnessed by Valentin, although carefully looked for in a great number of trials. Moreover, no movements can be produced in the diaphragm by irritating the vagus or sympathetic nerves; so that its action would seem to depend on the phrenic alone. There is reason to think that this nerve contains sensory as well as motor fibres; signs of pain being given when it is cut or compressed.

The Second Book is devoted to the Sympathetic system; the true nature of which is briefly discussed in the *first chapter.* Contrary to the opinion of most other physiologists, he does not regard it as a system complete in itself, but as entirely derived from the cerebro-spinal; its most prominent difference from the branches of that system being the repetition of its ganglia, so that the ganglionic influence is communicated to the fibres several times in their course. We may remark, however, that this distinction does not hold good in regard to the analogue of the cerebro-spinal system in articulated animals, in which there is a similar repetition of ganglionic centres—these being continuously united in the spinal cords of vertebrata. As in the ganglia of other nerves, so in those of the sympathetic, nucleated globules are seen, which are surrounded by sheaths of their own, and are either superposed on the bundles of fibres, or are dispersed through the plexus. Their sheaths are prolonged, with certain fibres peculiar to them, amongst the primitive nervous fibres proceeding from the ganglion. But, according to Valentin, these peculiar fibres are not nervous, as maintained by Remak and Müller.

CHAP. II. *On the Motor Functions of the Sympathetic Nerve.* These may be ascertained by experiment, not so well on the living body as on the dead body still irritable. They are dependent upon the fibres transmitted from the cerebro-spinal system; and they may be reduced to laws as definite as those which govern the movements of other parts of the body. But as the parts supplied by this nerve may be excited to motion otherwise than by a stimulus transmitted through it, experiments upon its functions are liable to the influence of many disturbing causes, and, to be satisfactory, must be frequently repeated. The results stated by Valentin have been obtained by experiments on more than three hundred animals—principally horses, sheep, and rabbits; dogs and cats being less employed, not because the experiments are the less successful in them, when performed under the same conditions, but because they very rapidly lose their irritability after death.

The first series of results relate to the *vascular system.* Valentin states that, with certain precautions which he enumerates, motions of the *heart,* more or less decided, may be excited by irritation of the roots of the spinal accessory nerve, and of the first four cervical nerves: also of the trunk of the par vagum, between the larynx and thorax; of the lowest cervical, and (in a slight degree) of the first thoracic ganglion of the sympathetic; and of the cardiac plexus. Hence it appears that the motor fibres of the last-mentioned nerves are derived from the first; and it is only when the cervical portions of the vagus and sympathetic are uninjured, that irritation of the roots of the spinal nerves produces any effect. He thinks that he has witnessed a distinct contraction of the thoracic aorta, the inferior cava, and the thoracic duct, upon irritation of the neighbouring portion of the sympathetic system; but upon this point he does not speak with certainty.

The second series refers to the *digestive system.* According to our author, the *pharynx* is strongly contracted on irritation of the roots of the accessory and first two cervical nerves, as well as of the pharyngeal branches of the vagus. The lower part of the *œsophagus* is made to contract peristaltically from above downwards by irritation of the cervical portion of the sympathetic in the rabbit, and of the sympathetic and vagus, which are united, in the dog and horse; also by irritation of the roots of the first three cervical nerves, and more rarely of the accessory. The thoracic portion of the œsophagus is made to contract by irritation of the lowest sympathetic ganglion of the neck, and of the higher cervical ganglia, of the œsophageal plexus, and of the roots of the lower cervical spinal nerves. Muscular contractions of the *stomach* are produced by irritation of the cervical part of the vagus, and also of its thoracic and abdominal portions; the lower the part of the nerve irritated, the nearer to the pylorus do the effects manifest themselves. Irritation of the roots of the fourth, fifth, sixth, and seventh cervical nerves and of the first thoracic in the rabbit produces very distinct contractions of the stomach, so that a complete furrow is evident between the cardiac and pyloric portion of the viscus; and the lower the nerve irritated, the nearer the pylorus do the contractions extend. Irritation of the first thoracic ganglion of the sympathetic produces the same effect. Hence it appears that the motor fibres of the stomach proceed from the four lowest cervical and the first (and in the sheep the second) thoracic nerves. Contractions of the *intestinal tube*, varying in place according to the part of the spinal cord experimented on, may be excited by irritation of the roots of the dorsal, lumbar, and sacral nerves, and of the trigeminus. In the horse and cat, a distinct peristaltic action of the small intestines has been seen on irritating the roots of the oculo-motor nerve; but this has not been noticed in the dog and rabbit, and in the cat a similar motion was once observed on irritating the roots of the accessory nerve. Similar effects to those obtained by experiments on the spinal nerves were produced by irritation of the lower part of the thoracic portion of the lumbar, and of the sacral portions of the sympathetic; also of the splanchnic branches, and of the gastric plexus. A peristaltic contraction, evident though slight, of the *ductus choledicus*, was produced by irritating the right splanchnic nerve (r. major).

The movements of the *respiratory system* are produced rather through the vagus than through the sympathetic nerve.

The motions of the *uropoiëtic system* may, like those of the alimentary canal, be excited by irritation of the roots of the spinal nerves, and of the sympathetic, which contains the fibres of these. A very distinct and powerful peristaltic action of the *ureter*, proceeding from the kidneys to the bladder, may be produced by irritating the abdominal ganglia, or the roots of the abdominal spinal nerves. Strong contractions of the *bladder* are excited by irritation of the inferior portion of the abdominal sympathetic, but especially of the sacral portion; and by irritation of the roots of the middle and inferior abdominal nerves of the spine. In these, as in former cases, no effect is produced by irritation of the spinal nerves, unless the portion of the sympathetic connected with the particular organ is entire.

Of the *genital system*, Valentin has found that contractions may be excited in the vas deferens and vesiculæ seminales, especially in the guinea-pig at the time of heat, by irritation of the inferior lumbar and highest sacral portions of the sympathetic; and also that contractions of the fallopian tubes and of the uterus itself, from the fundus to the neck, may be excited by irritation of the same nerves as those which excite the rectum, namely, the lower lumbar and first sacral nerves of the spine. This contraction was sometimes much less in the gravid than in the unimpregnated uterus.

The following inferences are drawn by Valentin from these experiments: 1. That all the parts which exhibit involuntary movement are excited by action, like voluntary muscles, by stimuli applied to the nerves with which they are endowed. 2. That from whatever part of the sympathetic system their nerves arise, their actions are governed by the same laws. 3. That the sympathetic system has the following relations with other nerves: *a*, its motor fibres are distributed to remoter parts of the body; *b*, still, throughout their long course, there is no connexion between them, so that definite contractions are excited according to the fibres irritated, as in other nerves; *c*, that these motor fibres originally proceed from the cerebro-spinal system, and that irritation of their origins acts through the sympathetic trunks.

It appears therefore that, although any particular division of the sympathetic nerve must be regarded as extremely complex in its character, deriving its motor fibres from many different sources, the ultimate distribution of these fibres is sufficiently simple, so that each organ is definitely supplied from a certain part of the cerebro-spinal axis. But the fibres proceeding from the roots of the cerebro-spinal nerves do not pass into the nearest organs, but are transmitted through three or more ganglia of the sympathetic, before being distributed on them. In this respect microscopic examination fully bears out the results of experiment. The great bulk of the fibres which go forth from one of the thoracic or abdominal ganglia come into it by the trunk with which it communicates with the one above; a few only originating in the ganglion itself.

The results of his own experiments are then applied by Valentin to elucidate the contradictory accounts of other physiologists, of which he gives a very complete summary. In doing this, he adopts a view subsequently developed more fully, that the movements of the heart, alimentary canal, &c. are ordinarily of a reflex character, and that they are not in the living animal excited by those *direct* stimuli by which they may be caused to take place in the dead body, when cut off from all nervous influence. On the merits of this doctrine we shall hereafter remark.

In regard to the *sensory* functions of the sympathetic nerve, discussed in the *third* chapter, Valentin states as the result of his experiments on the abdominal ganglia, that a slight irritation, whether mechanical or chemical, does not occasion pain immediately after the abdomen has been opened; but that a more powerful irritation, as by compression, ligature, or caustic, excites not only pain but vehement cries. The longer the ganglion has been exposed to the air (of course within certain limits) the more readily does irritation of it cause pain. The cords which pass out from the ganglia are not so readily excited by slight irritants as the branches which enter them, and the branches which communicate between the ganglia and the spinal nerves are sensory to such a degree that scarcely any difference can be perceived between them and the spinal nerves themselves. It is not as easily proved with regard to the sensory as with respect to the motor fibres of the sympathetic system, that they proceed from the spinal nerves; nevertheless, there can be little doubt that this is the case, as Valentin clearly shows by many arguments, and to these are due all the sensations which are referred to these parts in health or disease.

In the *fourth* chapter, the origin of the nervous fibres of the sympathetic is briefly treated of. These he considers, in opposition to the opinion of most anatomists, to proceed ultimately from the cerebro-spinal system; and he does not recognize the gray or organic fibres which are described by Remak and Müller. To this point we shall return hereafter, when we follow our author in his account of the nucleated globules. He states that the sympathetic in the neck is at first developed in the same manner as in the lower part of the trunk, each vertebra having a corresponding ganglion, but that a metamorphosis afterwards takes place, some of these disappearing and others being more fully evolved.

In the THIRD BOOK are discussed the laws which govern the actions of the peripheral nerves; and the *first chapter* is devoted to an exposition of their *physiological symmetry.* That of the spinal nerves is obvious enough, but that of the cranial is more recondite. Of these last, the vagus, spinal accessory, glosso-pharyngeus, and hypoglossus are to be regarded in the light of spinal nerves; and it is only, therefore, the first seven pairs that can be properly termed cerebral. The account given of their embryonic development is very curious and interesting, and throws great light upon their varieties of distribution in different classes. The three nerves of special sense arise, as has long been known, from the three vesicles which, at an early period, indicate the primary divisions of the encephalon. Afterwards, when the cranial vertebræ are developed, other nerves, intervertebral, are interposed among them. A separation of the primitive fibres of these, however, takes place, so that their distribution appears irregular. Thus the greater part of the sensory fibres are contained in the larger division of the trigeminus; whilst the motor seem to be chiefly transferred to the oculo-motor and patheticus. Again, of the primary anterior motor fibres, some form the small division of the trigeminus, whilst others unite with the first pair from the medulla oblongata to form the facial. Hence is understood the union of this nerve with those proceeding from the medulla oblongata in fishes and some amphibia. In regard to the medulla oblongata, which is gradually metamorphosed (as it were) from the medulla spinalis, it is to be noted that its nerves are all in the first instance double-rooted; but that the motor fibres of the first pair unite with those of the facial, whilst its sensory form the glosso-pharyngeal; whilst the second and third pairs become blended, the motor portion becoming the accessory and the sensory the par vagum. Our author lays much stress on the more complete metamorphosis or alienation from the character of spinal nerves, which may be traced in the anterior pairs of cerebral nerves, and on the gradual nature of this change, when traced upwards from the spinal cord, and he points out that, even of the special sensory nerves, the character of the olfactory is most unlike that of the spinal, whilst that of the auditory is most allied to it. He also notices that the relation of the olfactory with other sensory nerves is greater than that of the optic, and this last greater than in the auditory, and that the contrary is true regarding the motor fibres.

The division of the body into vertebræ or rings affects the formation and distribution of the nerves, as well as of other parts. According to Valentin (who thus confirms a statement formerly made on this point), the chain of sympathetic ganglia seems to be the first centre of the nervous system; the spinal nerves, or at least those which are subsequently to become so, rather coming off from the sympathetic than from the spinal cord. But, in process of time, the small cords by which they were connected with the spinal axis become the true roots, and those which proceeded from the sympathetic ganglia shrink into connecting branches. Some other very curious relations between the sympathetic and cerebro-spinal systems (for an account of which we must refer to the original) are unfolded by this kind of study. Those ganglia of the sympathetic system, with one of which every organ of sense is provided, are regarded by Valentin as having for their office to harmonise the motions of the external parts, and bring them in relation with the sensory actions of the organs themselves. Other kinds of symmetry in the nervous system are then pointed out; but these we may pass over, as of less special interest.

CHAP. II. *Of the Primitive Fibres.* That these, wherever they occur, serve only as conductors of the agency to which the nervous system ministers, can now scarcely be denied. This agency, of the actual nature of which we as yet know nothing, originates in the gray substance of the central organs, and in certain parts connected with the peripheral extremities of the nerves; and it depends on the peculiar relation of this system with the vascular. These two systems are denominated by Valentin *concreto-generalia*, being distinguished from other systems of the organism in this, that each forms, as it were, a whole, possessed of certain general properties, which undergo but slight modification in the several organs to which they are distributed, the peculiarity of *these* being rather adapted to *them*; so that the speciality of the actions depends upon the added or altered structure, and not upon anything peculiar in the operation of these systems. Such, at least, we understand to be Valentin's idea, which he refers to as more fully developed elsewhere. By the almost constant changes occurring in the nervous system, a continual action of some kind is maintained, both from the periphery towards the centre, and from the centre to the periphery; and this action seems to be intimately connected with the nutrition of the nerves themselves, since, if it be interrupted for any length of time, they lose their peculiar endowments. The peculiarity of the actions of the several kinds of fibres seems to depend more on the peculiarity of their terminations at the two extremities, than in anything in their own structure; and the specialities of sensory impressions are probably to be chiefly referred to the structure of the organs surrounding the peripheral terminations of the nerves. The influence of the conditions of the vascular systems on their functions is afterwards pointed out; and on this, as well as the foregoing points, his views completely correspond with those which we ourselves expressed not long since. (Vol. IX. p. 99.) A somewhat lengthy discussion of the primary changes, which are probably subservient to *objective* and *subjective* perceptions then follows; but this we shall not attempt to analyze, referring such of our readers who are interested in these abstruse metaphysico-physiological questions to the work itself. The general laws regarding the transmission of impressions along the nerves are also enumerated; but these, also, we shall pass by, as not containing anything of novel interest, though characterized by great clearness and caution in generalizing.

CHAP. III. *On the Formation of Ganglia.* That which essentially constitutes a ganglionic formation is the interposition of nucleated globules among primitive fibres, which have more or less of a plexiform arrangement. It is, therefore, erroneous to confound the *ganglionic* system with the *sympathetic*, since ganglionic formations occur in the cerebro-spinal system, as well as in this. A copious summary is given by Valentin of the opinions of authors as to the functions of ganglia, and he then proceeds to set forth his own, which proceeds upon the fundamental dogma (of the correctness of which we by no means feel satisfied), that the functions of the ganglionic structure are everywhere essentially the same. In the first place, he remarks, a ganglion does not produce any essential change in the endowments of the fibres which pass through it; sensory impressions are not reflected by them into motor impulses, but the branches which proceed from them contain a much greater admixture of fibres than those which enter. Ganglionic formations are found almost exclusively connected with sensory, or at least afferent nerves. The nucleated globules of the ganglia appear essentially to correspond with the globules of the gray substance. But the fibres of the central masses differ from those of the peripheral nerves in having their sheaths more slender and more similar to the coverings of the nucleated globules, which in the gray substance are so delicate that the globules themselves can with difficulty be distinguished, the whole mass having a tendency to form a soft colliquamentum. In the peripheral ganglia, however, the colliquamentum is firmer, the globules can be easily distinguished, their sheaths are thicker, and can be observed to send out processes amongst the primitive fibres; but there are among them several varieties, some of which indicate a near approach to those of the central system. Now, since the peripheral nucleated globules do not give rise to any actions of their own, it follows that their functions must be connected with those of the central system; and it may be inferred that they add strength to the operations of the nervous fibres which pass through them, especially to sensory impressions; perhaps, also, rendering the motor impulses of an automatic and periodic character more independent of the will. This general view of the actions of the peripheral ganglia is carried out into particulars with great ingenuity, but we cannot regard it as based on a solid foundation. The observations of Remak concur with those of Müller in leading to the conclusion, that the nucleated globules of the peripheral ganglia are related to a peculiar set of fibres, termed by them the gray or organic; which abound in the sympathetic system, constituting, indeed, its peculiar character, and which exist, in a less degree, in the cerebro-spinal, being mixed with them just as the fibres of the cerebro-spinal are mixed with the sympathetic. Admitting, therefore, all that Valentin has proved by experiment as to the influence of the cerebro-spinal system on the motions of the parts to which its fibres are distributed through the medium of the sympathetic, we cannot but think that this last has an independent action peculiar to itself (and to its ramifications scattered through the cerebro-spinal nerves), which we have on former occasions attempted to define. This is the portion of Valentin's treatise which seems to us least satisfactory.

Chap. iv. *On Reflex Action.* In this chapter we find a full summary of the facts established by the late researches of Dr. M. Hall, Müller, Volkmann, and others; which, having on former occasions pretty fully discussed, we shall not now revert to. In the opinion of Valentin, the reflexion of motions is not dependent upon sensations; the former being performed by the spinal cord or its segments independently of the brain which is the seat of sensibility. But he does not distinctly avow his belief in a separate system of fibres appropriated to the excito-motor or, as he denominates it, the *excitato-excitatorial* function; although from his previous statements respecting the distinctness of the endowments of individual fibres, it may be inferred that he holds that doctrine. He constantly speaks, however, of motor fibres being excited by *sensory;* which, on his own showing, is untrue. It is much to be wished that physiologists would abandon the use of this term, where the excitement of a sensation is not implied. The *direction* of the fibres,—afferent or efferent—centripetal or peripheral,—is in our minds a much better way of expressing their general endowments. According to Valentin, the motions of the heart and alimentary canal are ordinarily of a reflex character; and here sensation is certainly not excited. This, our readers will remember, is the doctrine long ago advocated by Whytt. That a motor impulse *may* be communicated from the spinal nerves to these muscles is, however, no proof to our minds that their actions are *dependent* upon it; and it only points out the channel through which, as every one knows to be the case, they are affected by particular states of mind, or by conditions of the general system. The performance of regular movements under the influence of direct stimuli, when these parts have long been separated from the nervous centres, and the analogy of these movements with those exhibited by plants (which we have formerly taken occasion to point out) are, in our opinion, quite sufficient evidence in favour of the Hallerian doctrine, especially when coupled with the fact, for such we esteem it, that the property of contractility is every-where inherent in muscular fibre, and that the stimulus of innervation is only *one* of the excitants by which it may be called into action.

In the Fourth Book is considered the influence of the peripheral nerves on the several functions. The *first* chapter is devoted to the special physiology of the senses; and in this a summary of our previous know-ledge of the subject is combined with many interesting views of the author's own; but these it would be difficult to present out of their connexion. One of the most interesting novelties is an extension of Weber's table of the distances at which two points can be separately distinguished in various parts of the surface of the body. Four additional sets of measurements by different individuals, varying in some particulars from Weber's and from each other, are given in a comparative form; and Valentin's own are added, with a column showing the mean of all.

Chap. ii. *On Motion.* After indicating the passive organs of locomotion, Valentin proceeds to consider the structure which actively contributes to the performance of this function—muscular fibre. He describes the two conditions in which it exists; in the muscles of voluntary motion and some others, the fibres being composite and marked with regular transverse striæ; whilst the muscular tissue of the alimentary canal and of the excretory ducts has the ultimate fibres separated and irregularly woven together. He then discusses the physiology of muscular action; and remarks very truly that no powerful contraction can be excited in muscular fibre, except by stimuli acting through the nervous system. But he does not thence infer, as many do, that the *contractility* of the muscle is dependent upon nervous action; but fully recognizes it as an inherent power, although in some way dependent for its continuance upon the integrity of the portion of the nervous centres with which the muscle is connected. On this point his views appear to correspond closely with those recently expressed by Dr. M. Hall. They are liable to the objection that, as Dr. J. Reid has shown, the irritability of a muscle after being exhausted may be recovered, even though deprived, by the section of its nerves, of all nervous agency; and there is no more difficulty in understanding why the peculiar property of a muscle should not be diminished and finally lost by the alteration in its nutrition consequent upon the cessation of its ordinary functions, than why the same should happen in other organs, as it is very well known to do. The investigation into the relations of nerve and muscle is of considerable length, and contains several facts of much physiological interest. A minute comparison is instituted between the composite and simple muscular fibres; but the general conclusion—that the former are more readily excited than the latter by stimuli acting through their nerves, and less readily by stimuli directly applied to themselves—does not differ from that ordinarily maintained. To Valentin, however, is due the merit of determining, with much more certainty than had been previously attained, that the simple muscular fibre *can be* excited to contraction through the nervous system. How far its ordinary actions are dependent upon such stimulation will be hereafter questioned.

A remarkable series of experiments on the brain and spinal cord of the frog then follows; having for their object to determine the connexion of their several parts with the movements of different organs of the body. The following are amongst the most interesting results: 1. In the frog, the motor fibres of the two sides of the medulla oblongata decussate just before they arrive at the fourth ventricle; so that the decussation affects those of the base of the cerebellum. 2. Neither the superior nor inferior strands (posterior and anterior columns in man) of the spinal cord solely possesses motor functions; but when the former are irritated sensations predominate, and when the latter, motions are chiefly excited. 3. If the *superior* column of the spinal cord be irritated at the point at which the nerves of either extremity are given off, that extremity is *extended;* if the *inferior* be irritated, the extremity is *flexed.* 4. Unless the irritation is excessive, or the will of the animal interferes, irritation of one side does not affect the other. At their entrance into the spinal cord, therefore, it would appear that the motor fibres of the extensors pass towards the superior stratum (posterior column in man), whilst those of the flexors are continuous with the inferior stratum; but their course is found to be altered, when they are examined at some distance from their point of exit. This doctrine is confirmed by experiments on mammalia; and by pathological phenomena in man. It fully explains the facts which led Bellingeri to maintain that the anterior roots of the nerves were for flexion, and the posterior for extension. By Valentin the Bellian view of the functions of the roots is rigorously upheld; and it is only in the account of the course of their fibres in the spinal cord, that he differs from the discoverer of their true endowments,

According to Valentin, relaxation of the sphincters is analogous to the extended state of the extremities; and he has noticed a relaxation of the sphincter ani in the frog, when the posterior part of the spinal cord was irritated so as to produce extension of the limbs. Further he states that the peristaltic and antiperistaltic motion of the intestines occurs under the same conditions respectively with the flexion and extension of the extremities.

The results of various experiments already recorded, on the relation of different parts of the nervous centres to muscular motion are then fully discussed; and lastly, the general laws of involuntary, automatic, and voluntary motion are laid down.

CHAP. III. *On Digestion.* In this chapter is considered the influence of the nervous system on the movements of the various parts of the alimentary canal, on which we have already sufficiently enlarged; and its influence upon the functions of secretion and assimilation. That the secretions necessary for digestion are not checked by section of the nerves has already been mentioned; and arguing from this, Valentin endeavours to make it appear that the functions of the solar plexus and its ramifications on the glandular system are confined to the maintenance of the movements of the alimentary canal, of the contractions of the ducts of the glands, and of the tonicity of the minute subdivisions of these.

CHAP. IV. *On the Circulation.* In this chapter Valentin attempts to prove that the rhythmical contractions of the heart are performed by *reflex* stimulation; and that the ganglionic plexus accompanying the vessels regulates their contractions. In reference to the first point he admits that the regular contractions of the heart removed from the body, continuing for a long time, and capable of being excited after they have ceased, is a considerable obstacle to his theory; in our minds it is an insuperable one.

CHAP. V. *On Respiration.* The several movements of respiration are discussed; and rightly attributed to reflex action, capable of being controlled by the will to a certain degree: according to our author, the nervous system has no direct influence on the chemical changes to which these movements are subservient.

CHAP. VI. *On the Preparation and Excretion of Urine.* This secretion affords great facilities to the experimenter, on account of the comparative facility with which all the nerves supplying the organ may be destroyed without interrupting the circulation. The results obtained by previous physiologists are collated by Valentin; and details of his own experiments are added. By these, and by pathological phenomena, he considers it proved that the secretion is not checked by the complete cessation of nervous influence; and that whatever change takes place in its character may be explained without the supposition that nervous agency has any direct influence on the act of secretion. Of the movements of the ureters and bladder an account has already been given.

CHAP. VII. *On Generation.* The formation of the seminal secretion is not influenced by the nervous system; but its ejaculation is as reflex movement excited through the spinal nerves. Erection of the penis and clitoris are regarded by Valentin as of a similar character; but though, when the action is commenced it may be increased by reflex stimulation of the muscles, we cannot regard the afflux of blood by which it is primarily effected as having any such cause. The movements of the fallopian tubes and of the uterus have been already noticed.

CHAP. VIII. *On the Nutrition and Reproduction of Parts.* The influence of the nervous system on these functions is not regarded by Valentin as any greater than on that of secretion. That they are affected by it is clearly proved by experiment and by pathological observation; but there is equally good reason to believe that they may go on, though with less than their normal regularity, when all nervous influence is cut off. He does not seem able to imagine that such nervous influence can consist in anything but the power of exciting motion; and it is, therefore, to the reflex actions of the blood-vessels, secretory ducts, &c., that he attributes the ordinary agency of the nerves upon these functions. He denies, as we have formerly hinted, the existence of those organic or gray fibres which have been described by Müller and Remak, as essentially constituting the sympathetic system. He conceives that the filaments, so denominated, are in reality extensions of the sheaths of the nucleated globules, which are formed of a sort of cellular tissue composed of primitive fibres interwoven together, and furnished with a kind of epithelium. This is not to us, however, by any means a satisfactory account of them; and we are much more disposed to believe, with Müller and Remak, that there is a distinct system of nervous fibres, to which the nucleated globules in the ganglia are the centres, specially connected with the organic functions, and designed to harmonise and blend these together, independently of any direct action of the cerebro-spinal system. That the great bulk of the so-called sympathetic nerve consists of fibres derived from the cerebro-spinal system, we can well believe, when it is considered how many are the influences exercised by particular states of mind upon the organic functions, and how close must be the connexion between these last and the animal functions which depend upon them for their continuance. And we think that it is rather too much a *mechanical* view to limit the operations of the nervous system to the production of motion. But in his general proposition that the sympathetic system of nerves does no more than direct and control the functions of nutrition, secretion, &c., the readers of this Journal need scarcely be informed that we fully participate; and it gives us much satisfaction to find this view adopted by so high an authority. The chapter concludes, and with this closes the volume, with an account of Valentin's own observations on the regeneration of nerves,—an occurrence of which he has fully satisfied himself, when the interval is not too large, and the trunk of sufficient size. In one instance a piece of the ischiatic nerve of the rabbit was cut out, to the extent of eight lines, and the injury was completely repaired after two or three months. The process of reproduction is very much what might have been predicted from observation of the embryonic development of the nervous system. At first the space is filled with lymph, which gradually becomes organized into cellular tissue. A yellowish oily substance is then seen to exude into this from the two ends of the nerve; and it permeates the cellular structure in both directions, so as to meet midway between the ends. Around its particles, which finally become white, the fibres of the cellular tissue are seen to arrange themselves,—at first separating it, as it were, into fasciculi,—and afterwards into what become the primitive nervous fibres.

Having thus brought our analysis to a conclusion, we would again express our conviction of the great importance of this work, and of the high rank which we think it ought to take amongst physiological treatises. We have never met with a work on any subject in which so much original matter of sterling value is so judiciously mixed up with the knowledge previously obtained from other sources. With all but the most recent enquiries prosecuted in this country (of which some of the most important have been made public subsequently to the completion of his labours), he shows himself well acquainted. We have thought it right to indicate, as we have proceeded, the chief points on which we differ from him; and these rather concern matters of mere speculation, than direct deductions from experiment and observation. There are many of his results which we should be glad to see confirmed in this country; but, when once fully established, we trust that no superfluous animal suffering will be inflicted, for the mere purpose of *exhibiting* them. There are still questions enough to be decided, to require as large a sacrifice of dogs and rabbits as the humane physiologist will feel at ease in offering upon the altar of science.

---

